# Multiscale Cortical Remodeling Following Abrupt Visual Deafferentation in Rhegmatogenous Retinal Detachment: Imaging Transcriptomics and Neurotransmitter Mapping

**DOI:** 10.1002/cns.70925

**Published:** 2026-05-13

**Authors:** Yu Ji, Xin Huang, Yuan‐Yuan Wang, Lin Zhou, Zhuo‐Er Dong, Xiao‐Rong Wu

**Affiliations:** ^1^ Department of Ophthalmology The First Affiliated Hospital, Jiangxi Medical College, Nanchang University Nanchang Jiangxi China; ^2^ The Affiliated Eye Hospital, Jiangxi Medical College, Nanchang University Nanchang Jiangxi China; ^3^ Jiangxi Province Key Laboratory of Ophthalmology and Vision Sciences Nanchang Jiangxi China; ^4^ Jiangxi Clinical Research Center for Ophthalmic Disease Nanchang Jiangxi China; ^5^ Department of Radiology The First Affiliated Hospital, Jiangxi Medical College, Nanchang University Nanchang Jiangxi China

**Keywords:** imaging transcriptomics, intrinsic neural timescale, Mendelian randomization, neurotransmitter density maps, rhegmatogenous retinal detachment, structural covariance network gradient

## Abstract

**Background:**

Neuroimaging evidence indicates brain alterations in rhegmatogenous retinal detachment (RRD), but the molecular and neurochemical correlates of these macroscale patterns remain unclear.

**Methods:**

We examined gray matter volume (GMV), intrinsic neural timescale (INT) and structural covariance network (SCN) gradients in 51 patients with RRD and 45 healthy controls (HCs). Two‐sample Mendelian randomization (MR) evaluated whether RRD‐related genetic liability was consistent with a putative causal effect on GMV. SCN‐gradient alterations were further evaluated using exploratory spatial association analyses based on the Allen Human Brain Atlas (AHBA) and complementary neurotransmitter maps. SHAP‐explainable machine‐learning classification models compared the discriminative utility of structural versus functional features.

**Results:**

Patients with RRD showed reduced GMV in the visual network (VN) and shortened INT in the default mode network (DMN). MR results were consistent with a putative causal effect of RRD genetic liability on VN atrophy. SCN gradients revealed a hierarchical “visual‐downward and limbic‐upward” shift. Exploratory imaging‐transcriptomic spatial association analyses suggested that gradient alterations were spatially aligned with gene expression patterns enriched for neurodevelopmental and synaptic pathways, including excitatory/inhibitory neuronal and microglial signatures; complementary neurotransmitter mapping analyses further suggested spatial correspondence with normative monoaminergic/cholinergic and μ‐opioid maps, together with an inverse spatial association with GABAa receptor density maps. INT‐based ML models outperformed GMV‐based models (best AUC = 0.753), with SHAP identifying INT as the predominant contributor.

**Conclusion:**

RRD is associated with coordinated structural and functional alterations across cortical hierarchies. Exploratory transcriptomic and neurotransmitter spatial association patterns may provide biological context for these imaging abnormalities and inform future studies of prognosis and underlying mechanisms.

## Graphical abstract

RRD triggers multiscale cortical reorganization, including visual‐network atrophy, shortened intrinsic neural timescale, and altered structural covariance gradients. Integrating Mendelian randomization with AHBA and PET/SPECT biological anchoring and SHAP‐based classification highlights INT as a sensitive imaging biomarker for RRD‐related brain remodeling.

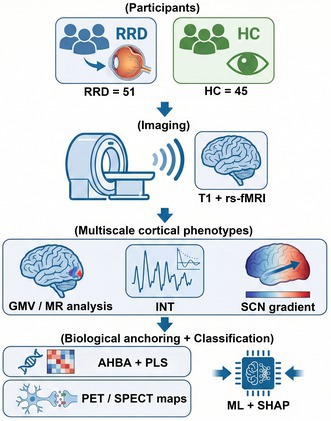

## 1 Introduction

Rhegmatogenous retinal detachment (RRD) is an acute ophthalmic disorder caused by retinal breaks, leading to separation of the neurosensory retina from the retinal pigment epithelium [1]. Patients typically present with floaters, photopsia, and visual field defects, and without timely treatment the disease may progress to profound and irreversible vision loss [2, 3, 4]. The global incidence of RRD is increasing, currently estimated at ~12.2 per 100,000 individuals (≈1 per 10,000) [5, 6]. Surgical repair—pars plana vitrectomy (PPV), scleral buckling (SB), or pneumatic retinopexy (PnR)—achieves high anatomical success [7, 8, 9], yet a considerable proportion of patients continue to experience persistent visual dysfunction, likely reflecting irreversible photoreceptor injury and residual visual field impairment [10]. Importantly, accumulating neuroimaging evidence indicates that RRD‐related visual deficits may extend beyond the retina, involving central visual pathways and higher‐order cortical/limbic regions associated with visual processing [11, 12, 13]. These findings suggest that RRD may involve cortical alterations following abrupt sensory input disruption, underscoring the need to clarify central changes associated with the disease.

Recent neuroimaging studies have reported widespread structural and functional abnormalities in RRD across gray and white matter [14, 15, 16, 17]. However, most work has focused on isolated regions or single modalities, limiting a system‐level understanding of structural–functional reconfiguration. Here, we examined complementary macroscale imaging phenotypes to characterize cortical alterations associated with RRD. Structurally, voxel‐based morphometry (VBM) provides voxel‐wise quantification of gray matter alterations [18], and gray matter volume (GMV) is commonly used as an MRI‐derived structural phenotype linked to regional cellular architecture [19]. Functionally, intrinsic neural timescale (INT) captures the temporal integration window of local neural activity [20, 21] and follows a cortical hierarchy from transmodal association regions (longer timescales) to primary sensorimotor systems (shorter timescales) [22, 23], with evidence for coupling to structural properties [24, 25]. At the organizational level, the cortex is arranged along a hierarchical “sensory–fugal” axis that transitions from unimodal to transmodal systems [26]. Gradient mapping based on diffusion embedding enables low‐dimensional representations of continuous topographic transitions across the cortex [27]. In this context, the structural covariance network (SCN), defined by inter‐regional covariance of morphological features [28], provides a useful framework for characterizing macroscale structural alterations across development and disease [29, 30, 31]. To address this methodological gap, we integrated GMV, INT, and SCN gradients to systematically characterize multiscale cortical alterations associated with RRD and examine whether abrupt visual deafferentation is associated with coordinated structural and functional alterations.

Beyond macroscale imaging, linking brain phenotypes to molecular and genetic architectures can provide biologically informative context. Gene expression resources bridge transcriptional patterns with large‐scale brain organization [32, 33, 34], and the Allen Human Brain Atlas (AHBA) enables spatial correspondence analyses between regional imaging signatures and genome‐wide expression profiles [35, 36, 37]. Neurotransmitters, key mediators of synaptic signaling [38], show heterogeneous spatial distributions that shape neural states and plasticity and may constrain large‐scale network organization [39]. Advances in PET/SPECT tracers have enabled quantitative mapping of neurotransmitter and receptor distributions [40, 41], and recent work has integrated MRI‐derived phenotypes with PET/SPECT maps to illuminate neurochemical influences on brain organization [42, 43, 44]. In parallel, genome‐wide association studies (GWAS) provide genetic instruments for causal inference [45, 46], and Mendelian randomization (MR) leverages lifelong stable allelic variation to estimate causal effects while mitigating confounding [47, 48]. Building on these approaches, we incorporated AHBA transcriptomics, neurotransmitter distribution maps, and MR analyses to explore the population‐level molecular and neurochemical context of RRD‐related cortical alterations.

Machine learning (ML) offers additional sensitivity for detecting distributed neuroimaging signatures [49, 50], although clinical translation requires interpretability [51]. The SHapley Additive exPlanations (SHAP) framework quantifies feature contributions to model outputs, enhancing transparency and biological interpretability [52, 53]. Accordingly, as an auxiliary analysis, we employed five complementary classifiers—support vector machine (SVM), random forest (RF), logistic regression (LR), extreme gradient boosting (XGBoost), and light gradient boosting machine (LightGBM)—to evaluate the discriminative value of GMV and INT features between RRD and healthy controls (HCs), and used SHAP to identify the most influential imaging markers.

**FIGURE 1:**
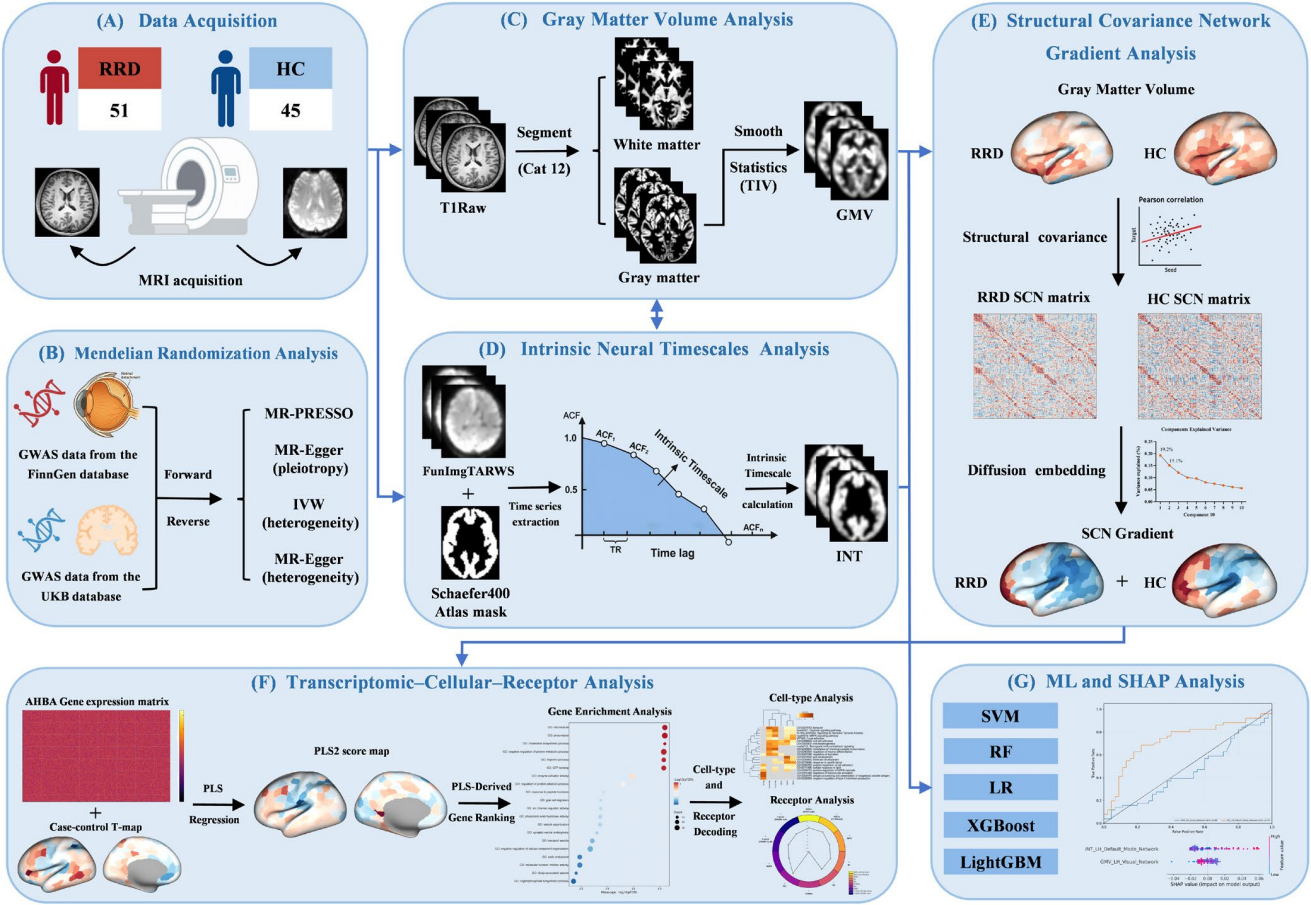
Study workflow. MRI‐derived GMV, INT, and SCN gradients were integrated with bidirectional MR, imaging transcriptomics (AHBA/PLS), neurochemical mapping, and interpretable machine‐learning classification (SHAP) to characterize multiscale cortical remodeling in RRD. AHBA, Allen Human Brain Atlas; GMV, gray matter volume; HCs, healthy controls; INT, intrinsic neural timescale; MR, Mendelian randomization; PLS, partial least squares; RRD, rhegmatogenous retinal detachment; SCN, structural covariance network; SHAP, SHapley Additive exPlanations.

We hypothesized that RRD is associated with spatially distributed structural and functional alterations, and further examined whether these imaging patterns show spatial correspondence with regional gene expression and neurotransmitter architectures. To test this hypothesis, we first quantified GMV alterations using T1‐weighted MRI and performed MR using large‐scale GWAS data to assess potential causal links between RRD liability and GMV. We then computed INT from resting‐state fMRI to evaluate temporal integration properties and constructed SCN gradients based on GMV to characterize macroscale hierarchical shifts. Next, we performed exploratory imaging–transcriptomic and neurotransmitter spatial mapping analyses to examine whether these imaging alterations show spatial correspondence with population‐level molecular and neurochemical reference maps. Finally, we applied multiple ML classification models with SHAP as an auxiliary analysis to assess classification performance and identify key discriminative features. The overall workflow is summarized in Figure 1.

## 2 Participants and Methods

### 2.1 Participants

This study was approved by the Ethics Committee of the First Affiliated Hospital of Nanchang University (Approval No. IIT [2024] Ethics No. 790), and written informed consent was obtained from all participants. A total of 96 individuals were included (RRD, *n* = 51; HCs, *n* = 45). RRD was independently confirmed by two retinal specialists using fundus examination, optical coherence tomography (OCT), and B‐scan ultrasonography. Groups were matched for age, sex, and years of education. Detailed eligibility criteria and screening procedures are provided in Appendix S1.

### 2.2 MRI Data Acquisition

MRI data were acquired at the First Affiliated Hospital of Nanchang University on a 3.0‐T Siemens Trio Tim scanner using an eight‐channel phased‐array head coil. High‐resolution T1‐weighted images and resting‐state fMRI were collected with participants awake and eyes closed. Detailed acquisition parameters are provided in Table S1.

### 2.3 VBM Analysis

Voxel‐based morphometry was performed using CAT12 implemented in SPM12 (MATLAB R2022b). T1‐weighted images were segmented and normalized to Montreal Neurological Institute (MNI) space, and modulated gray matter maps were smoothed with an 8‐mm full‐width at half‐maximum (FWHM) Gaussian kernel. Group differences in GMV were tested using two‐sample *t*‐tests with age, sex, and total intracranial volume as covariates, with Gaussian random field (GRF) (voxel *p* < 0.01; cluster *p* < 0.01). Detailed preprocessing and quality control procedures are provided in Appendix S1.

### 2.4 MR Analysis

Two‐sample Mendelian randomization (MR) was performed to test whether genetic liability to retinal detachment–related phenotypes (FinnGen R9) was associated with regional GMV (UK Biobank imaging GWAS; *n* = 33,224, European ancestry). Genome‐wide significant variants (*p* < 5 × 10^−6^; *F* > 10) were used as instrumental variables. Causal effects were estimated using IVW (multiplicative random effects), with MR‐Egger, weighted median, and mode‐based estimators as sensitivity approaches. Heterogeneity, horizontal pleiotropy, and outliers were assessed using Cochran's *Q*, the MR‐Egger intercept, and MR‐PRESSO. Additional details (exposure definitions, instrument selection, and sensitivity analyses) are provided in Appendix S1.

### 2.5 INT Analysis

Resting‐state fMRI data were preprocessed using a standard pipeline (details in Appendix S1). INT was computed within the Schaefer‐400 atlas as the area under the initial positive segment of the voxel‐wise autocorrelation function of the BOLD signal, scaled by repetition times (TR), yielding whole‐brain INT maps for each participant. Group differences were assessed using two‐sample *t*‐tests with GRF correction (voxel *p* < 0.01; cluster *p* < 0.01, two‐tailed).

### 2.6 SCN Gradient Calculation

SCN gradients were derived from group‐level structural covariance matrices constructed separately for RRD and HCs after regressing out age, sex, and mean GMV. Partial Pearson correlations between parcel‐wise GMV values were Fisher z‐transformed and decomposed using diffusion embedding (BrainSpace) [54]. The covariance matrices were thresholded at 90% sparsity and converted to affinity matrices using a normalized angle kernel (*α* = 0.5) [27, 55]. Gradient spaces were aligned across groups using Procrustes rotation. Additional details and reproducibility analyses are provided in Appendix S1.

### 2.7 Gene Expression Data Preprocessing

AHBA microarray data from six adult donors were processed using the abagen toolbox [56] and mapped to the Schaefer‐400 parcellation following established workflows. Analyses were restricted to the left hemisphere due to limited right‐hemisphere sampling in the AHBA. After preprocessing, 15,633 genes were retained, yielding a parcel‐wise expression matrix used for subsequent imaging–transcriptomic analyses. Detailed preprocessing steps and donor information are provided in Appendix S1 and Table S2.

### 2.8 Transcription–Neuroimaging Association Analysis

Partial least squares (PLS) regression [57] was used to relate parcel‐wise AHBA gene expression to the group‐difference map of SCN gradient 1. Component significance was assessed using a spatial spin permutation test (10,000 permutations). Gene weights were evaluated with permutation testing and false discovery rate (FDR) correction (*p* < 0.05), and significant genes were carried forward for enrichment analyses. Additional implementation details are provided in Appendix S1.

### 2.9 Gene Enrichment Analysis

Functional enrichment analysis was performed for genes with significant PLS weights using Metascape [58], focusing on GO terms (BP, MF, and CC). Enrichment was assessed with FDR correction (*p* < 0.05) and conducted separately for positively and negatively weighted gene sets. Full results are provided in Appendix S1.

### 2.10 Cell Type Analysis

Cell‐type specificity of genes identified from the PLS analysis was evaluated by testing overlap with curated marker gene sets derived from large‐scale single‐cell transcriptomic studies of the human cortex. Seven major cell types were examined (astrocytes, endothelial cells, microglia, excitatory neurons, inhibitory neurons, oligodendrocytes, and oligodendrocyte precursor cells). Significance was assessed using permutation testing with FDR correction (*p* < 0.001). Additional details are provided in Appendix S1.

### 2.11 Spatial Correlation With Neurotransmitter Density Maps

Spatial correlations between the SCN gradient‐1 case–control map and 44 PET/SPECT neurotransmitter receptor/transporter density maps were examined using the JuSpace toolbox [59]. Significance was assessed using 1000 spatial permutations for each map, with FDR correction across all maps; full atlas details are provided in Appendix S1.

### 2.12 Machine Learning Analysis

Significant GMV and INT features were used to train five binary classifiers (SVM, RF, LR, XGBoost, and LightGBM) to distinguish RRD patients from HCs. Model performance was evaluated using repeated nested cross‐validation, with an outer 10‐fold stratified cross‐validation repeated 10 times for performance estimation and an inner fivefold grid search for hyperparameter optimization based on the area under the receiver operating characteristic curve (AUC). Feature standardization was implemented within a pipeline and fitted only on the training data in each fold to reduce data leakage. Classification performance was quantified using accuracy, AUC, sensitivity, specificity, precision, and F1‐score. To determine whether the observed performance exceeded chance level, 5000‐label permutation tests were additionally performed. Further implementation details are provided in Appendix S1.

### 2.13 SHAP Analysis

To provide post hoc interpretability of the classification models, SHAP was used to estimate the contribution of each feature to model output. TreeExplainer was applied to tree‐based models (RF, XGBoost, and LightGBM), whereas KernelExplainer was used for non‐tree models (SVM and LR). Further implementation details are provided in Appendix S1.

### 2.14 Statistical Analysis

Normality was assessed using the Shapiro–Wilk test. Demographic/clinical variables were compared using *t*‐tests or Mann–Whitney *U* tests as appropriate, and *χ* ^2^ tests for categorical variables. Neuroimaging group comparisons used two‐sample *t*‐tests with multiple‐comparison correction as specified for each analysis: age, sex, and years of education were included as covariates. Additional implementation details are provided in Appendix S1.

## 3 Results

### 3.1 Demographic Characteristics

Fifty‐one patients with RRD and 45 HCs were included. Age and sex did not differ between groups (Table S3).

### 3.2 Group Differences in GMV

**FIGURE 2:**
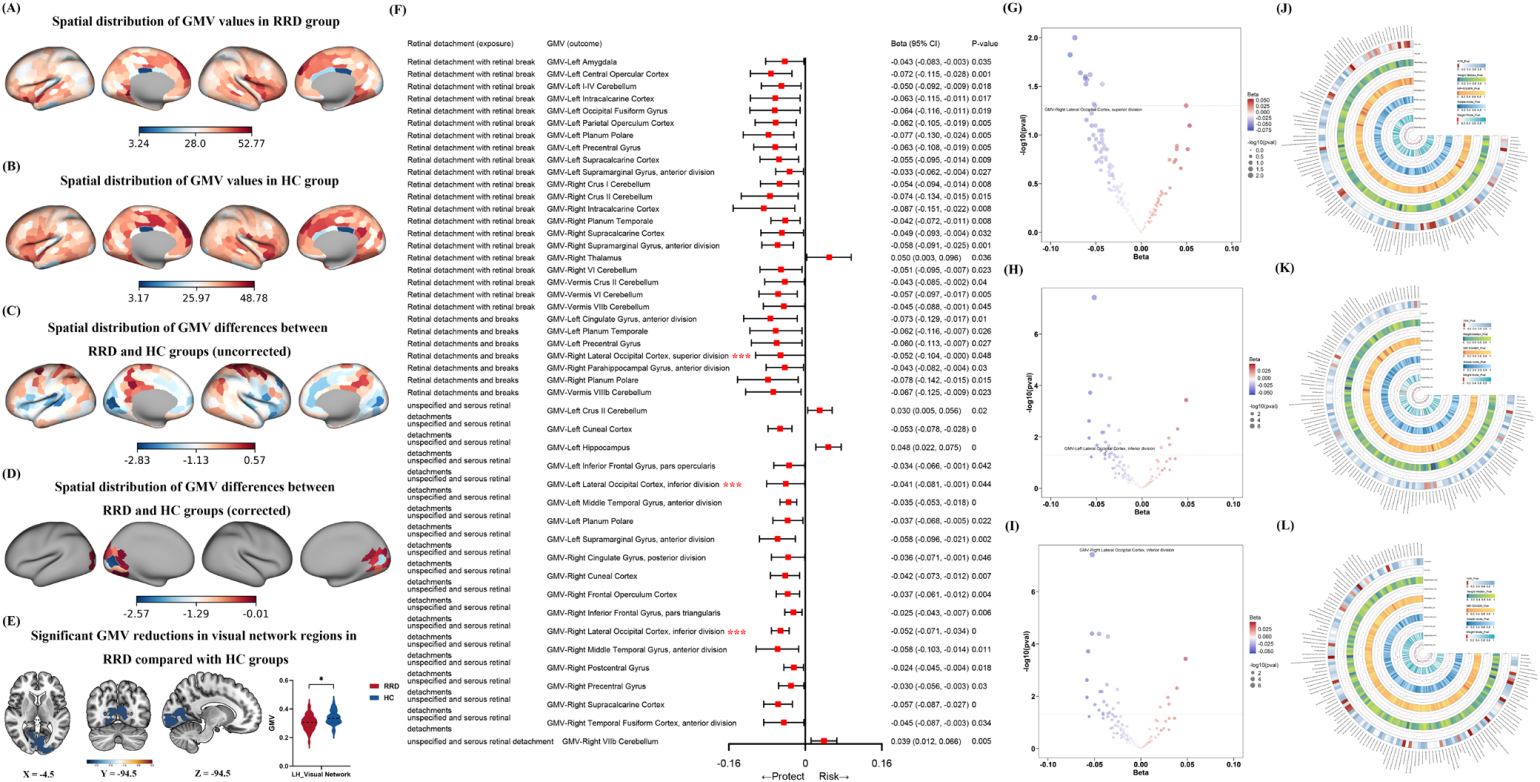
GMV reduction in RRD and causal links to retinal detachment liability. VBM revealed decreased GMV in RRD versus HCs after GRF correction (A–E; voxel *p* < 0.01; cluster *p* < 0.01; two‐tailed; Table S4). The asterisk (*) in panel E indicates the statistically significant GMV reduction in RRD compared with HCs after GRF correction. Forward MR showed significant associations between retinal detachment–related phenotypes and lateral occipital GMV, with MR–GMV overlap highlighted (F–L). MR details are reported in Table S5A–F. GMV, gray matter volume; GRF, Gaussian random field; HCs, healthy controls; MR, Mendelian randomization; RRD, rhegmatogenous retinal detachment.

Compared with HCs, patients with RRD showed significantly reduced GMV in the left visual network (LH_Visual_Network; GRF‐corrected voxel‐level *p* < 0.01, cluster‐level *p* < 0.01, two‐tailed; Figure 2D,E). Peak statistics and cluster size are provided in Table S4.

### 3.3 Causal Relationships

Forward MR identified three significant exposure–outcome pairs involving the lateral occipital cortex, which overlapped with the VBM‐derived GMV reduction pattern (Figure 2F–L). Sensitivity analyses were negative (all *p* > 0.05), and full instruments/estimates for forward and reverse MR are provided in Table S5A–F.

### 3.4 Group Differences in INT

**FIGURE 3:**
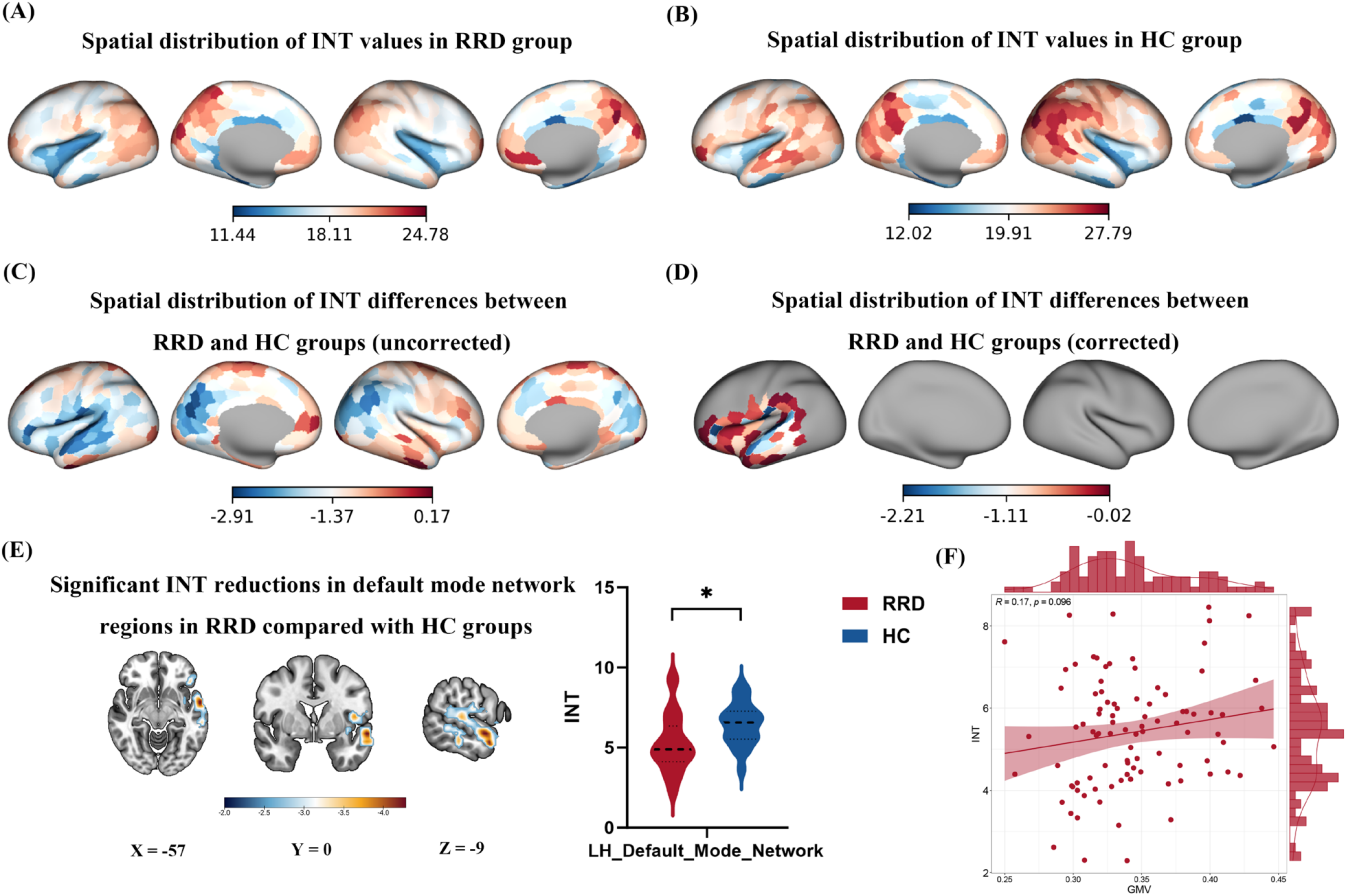
Reduced INT in RRD. RRD showed decreased INT relative to HCs after GRF correction (A–E; voxel *p* < 0.01; cluster *p* < 0.01; two‐tailed; Table S4). The asterisk (*) in panel E indicates a statistically significant group difference after GRF correction. Mean GMV and INT were not significantly correlated (F). GMV, gray matter volume; GRF, Gaussian random field; HCs, healthy controls; INT, intrinsic neural timescale; RRD, rhegmatogenous retinal detachment.

Compared with HCs, patients with RRD showed significantly reduced INT in the left default mode network (LH_Default_Mode_Network) after GRF correction (voxel‐level *p* < 0.01; cluster‐level *p* < 0.01; two‐tailed; Figure 3D,E). Peak statistics and MNI coordinates are reported in Table S4. Mean GMV and mean INT were not significantly correlated across participants (Spearman *r* = 0.17, *p* = 0.096; Figure 3F).

### 3.5 SCN Gradient Results

**FIGURE 4:**
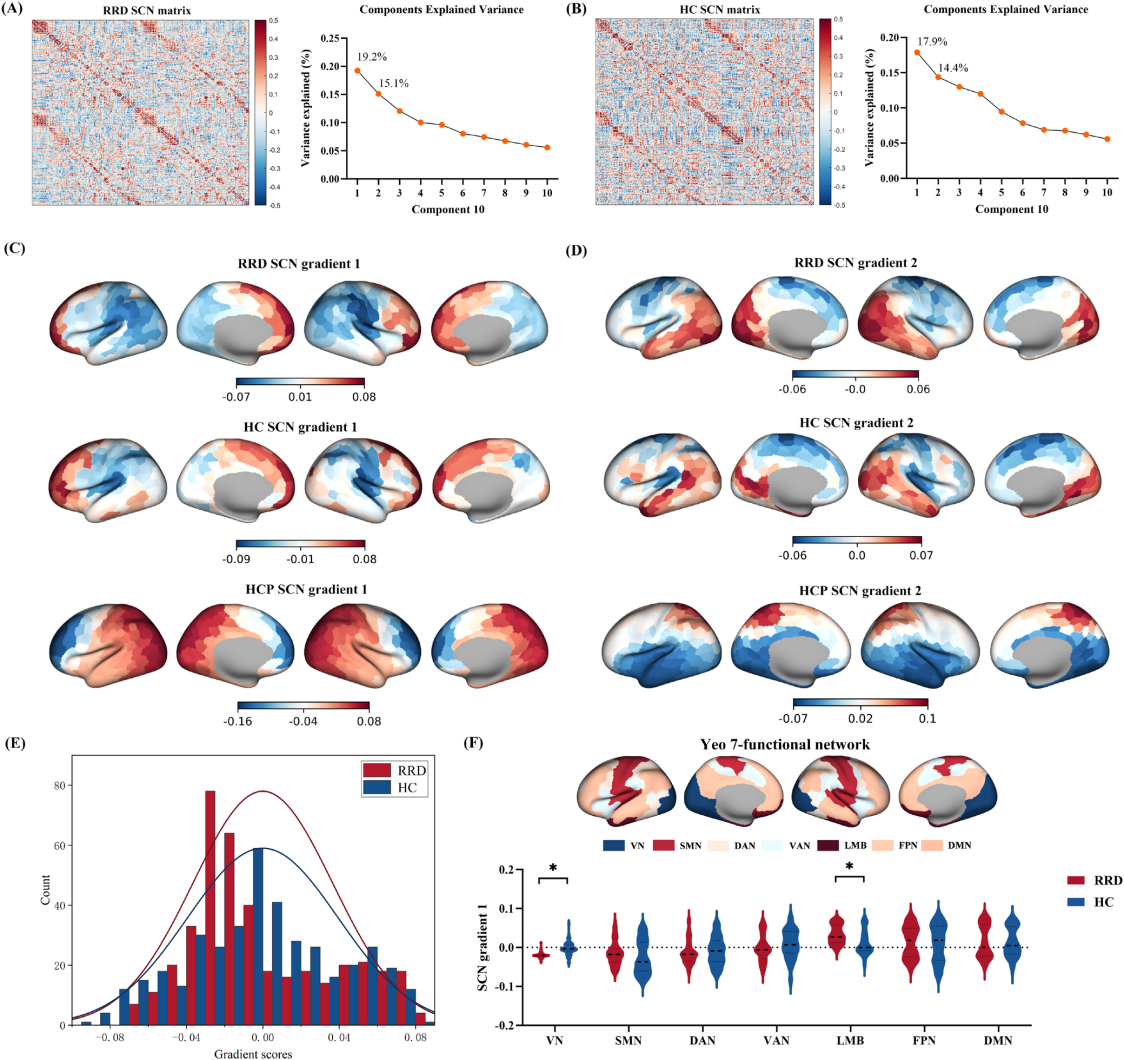
SCN gradients in RRD and HCs. GMV‐based SCNs and variance explained by the first 10 components are shown for RRD and HCs (A, B); Gradient 1/2 explained 19.2%/15.1% (RRD) and 17.9%/14.4% (HCs). Whole‐brain maps depict SCN Gradients 1 and 2 in RRD, HCs, and the HCP reference (C, D). The distribution of Gradient 1 scores differed between groups (E), and Yeo‐7 network–wise comparisons revealed significant between‐group differences in the visual (VN) and limbic (LMB) networks (F). The asterisks (*) in panel F indicate FDR‐corrected significant between‐group differences. HCP, Human Connectome Project; HCs, healthy controls; LMB, limbic network; RRD, rhegmatogenous retinal detachment; SCN, structural covariance network; VN, visual network.

SCNs were constructed from GMV separately for RRD and HCs, thresholded at 90% sparsity, and decomposed to obtain 10 gradient components. Gradients from the RRD group were aligned to the HC reference (Figure 4A–D). Gradient 1 explained 19.2% (RRD) and 17.9% (HCs) of variance, whereas Gradient 2 explained 15.1% (RRD) and 14.4% (HCs). Gradient 1 showed significant spatial correspondence between HCs and the Human Connectome Project (HCP)‐derived Gradient 1 (*r* = 0.089, *p*_spin < 0.05), while Gradient 2 did not (*r* = −0.456, *p*_spin > 0.05); therefore, subsequent analyses focused on Gradient 1.

Group distributions of Gradient 1 scores differed between RRD and HCs (Figure 4E). Across Yeo‐7 networks, Gradient 1 scores showed significant between‐group differences after FDR correction in the visual network (VN; *t* = −7.570, *p* < 0.001) and limbic network (LMB; *t* = 4.833, *p* < 0.001) (Figure 4F).

### 3.6 Transcription–Neuroimaging Associations

**FIGURE 5:**
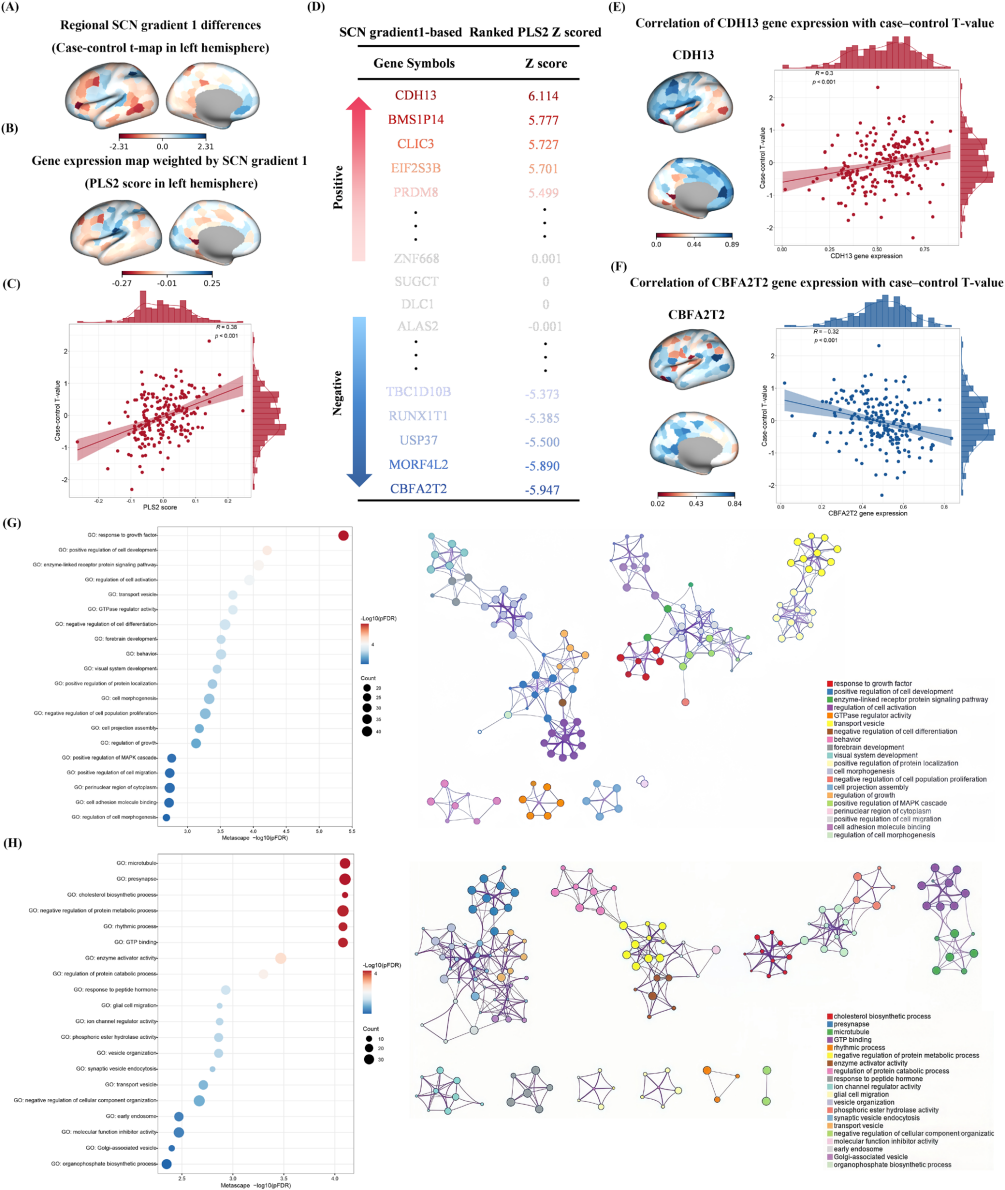
Imaging transcriptomics and functional enrichment linked to SCN Gradient 1 abnormalities. PLS regression related the SCN Gradient 1 case–control t‐map to AHBA gene expression (A–C) and identified a PLS2 component with positively and negatively weighted genes (D), with CDH13 and CBFA2T2 shown as representative examples (E, F). GO enrichment (Metascape) revealed distinct functional signatures for PLS2+ and PLS2− gene sets (G, H). Full PLS‐derived gene lists and *Z*‐scores are provided in Table S6A,B. AHBA, Allen Human Brain Atlas; GO, Gene Ontology; HCs, healthy controls; PLS, partial least squares; RRD, rhegmatogenous retinal detachment; SCN, structural covariance network.

To link SCN Gradient 1 abnormalities to transcriptomic architecture, we performed PLS regression between AHBA gene expression and the case–control t‐map of Gradient 1 differences (Figure 5A). The resulting PLS2 gene‐weighted expression scores showed a spatially ordered cortical pattern (Figure 5B). PLS2 explained 16.35% of variance and was significant relative to spatial permutations (*p*_spin < 0.05; *r* = 0.376), and PLS2 scores correlated with the Gradient 1 t‐map (*r* = 0.380, *p* < 0.001; Figure 5C). After FDR correction (*p* < 0.05), 749 positively weighted genes (*Z* > 2.84) and 663 negatively weighted genes (*Z* < −2.84) were identified (Figure 5D), with CDH13 and CBFA2T2 shown as representative examples (Figure 5E,F). The full gene lists and *Z*‐scores are provided in Table S6A,B.

### 3.7 Enrichment Pathways of Genes Associated With Changes in SCN Gradient

GO enrichment analysis (Metascape) of the PLS2‐weighted gene sets revealed distinct functional signatures (Figure 5G,H). PLS2+ genes were enriched for growth factor response and neurodevelopment‐related programs, including regulation of differentiation and morphogenesis. In contrast, PLS2− genes were enriched for neuronal structural and presynaptic terms (e.g., microtubule/presynapse), as well as pathways related to protein turnover and membrane remodeling. Together, these results suggest that SCN Gradient 1–related transcriptomic alterations reflect a balance between developmental regulatory processes and synaptic structural maintenance.

### 3.8 Transcriptional Signatures for Canonical Cell Types

**FIGURE 6:**
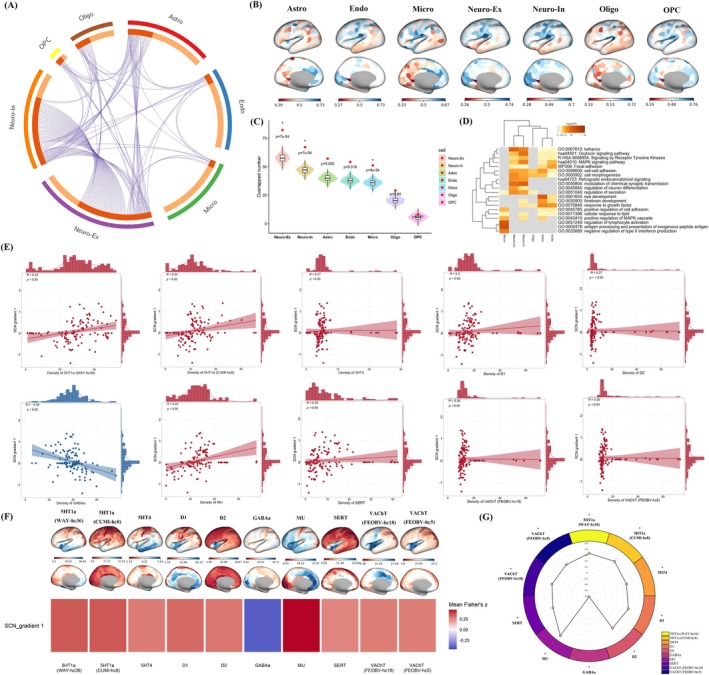
Cell‐type and neurotransmitter mapping of SCN Gradient 1 abnormalities. PLS2+ genes were enriched mainly in neuronal cell types (with additional microglial enrichment) (A–D), and SCN Gradient 1 abnormalities showed significant correspondence with PET/SPECT neurotransmitter maps (E–G). HCs, healthy controls; PET/SPECT, positron emission tomography/single‐photon emission computed tomography; PLS, partial least squares; RRD, rhegmatogenous retinal detachment; SCN, structural covariance network.

Cell‐type enrichment mapping across seven canonical cortical cell classes showed significant enrichment only for the PLS2+ gene set (Figure 6). PLS2+ genes were preferentially enriched in neuronal populations (Neuro‐Ex and Neuro‐In), with additional enrichment in microglia (Micro), whereas oligodendrocyte‐related classes (Oligo and OPC) showed the weakest signals (Figure 6A–C). Quantitatively, enrichment was significant for Neuro‐Ex (*n* = 82, FDR *p* < 0.001), Neuro‐In (*n* = 67, FDR *p* < 0.001), and Micro (*n* = 51, FDR *p* < 0.001). Functional annotations indicated that neuron‐enriched genes mapped to synaptic transmission and neurodevelopmental programs, whereas microglia‐enriched genes mapped to immune activation and inflammatory regulation (Figure 6D).

### 3.9 SCN Gradient and Neurotransmitter Distribution

The SCN Gradient 1 group‐difference map showed significant spatial correspondence with multiple neurotransmitter/receptor density maps (FDR‐corrected *p* < 0.001; Figure 6E–G). Positive correlations were observed for serotonergic (5‐HT1a/5‐HT4, SERT), dopaminergic (D1/D2), μ‐opioid (MU), and cholinergic (VAChT) systems, whereas correspondence with GABAa was weakest.

### 3.10 ML and SHAP Results

Across classifiers, models using INT_LH_Default_Mode_Network outperformed those using GMV_LH_Visual_Network (Table S7; Figure S1). The best performance was achieved by SVM with INT_LH_Default_Mode_Network (AUC = 0.753, accuracy = 0.688, F1 = 0.691). SHAP analysis consistently identified INT_LH_Default_Mode_Network as the dominant contributor to model predictions, with substantially higher mean absolute SHAP values than GMV_LH_Visual_Network (Figure S1).

## 4 Discussion

To the best of our knowledge, this is the first study to jointly examine GMV, MR, INT, and SCN gradient analyses in RRD, and to further relate these macroscale alterations to transcriptomic patterns, cell‐type enrichment, and neurotransmitter receptor distributions. We observed (i) reduced GMV in the left visual network with MR evidence supporting a putative causal effect of retinal detachment on visual cortical atrophy; (ii) shortened INT in the left default mode network (DMN); and (iii) significant Gradient 1 shifts across the visual and limbic modules, suggesting alterations in cortical hierarchy. Transcriptome–neuroimaging correspondence further suggested spatial associations with gene sets enriched for neurodevelopmental, synaptic, and metabolic pathways, together with population‐level cellular and neurochemical reference signatures, while machine learning showed superior performance for INT‐based models, with SHAP consistently identifying INT as the dominant contributor to classification, supporting its utility for interpretable individualized modeling.

At the structural level, RRD patients exhibited a marked GMV reduction predominantly within the left visual network, and MR analysis supported a causal effect of retinal detachment on GMV alterations in the visual cortex. As a hierarchical system centered on early occipital visual cortex [60], the VN appears particularly vulnerable to sustained deafferentation: convergent evidence across RRD, glaucoma, age‐related macular degeneration, and inherited retinal dystrophies consistently shows visual cortical GMV reductions that map onto regions of visual field loss [61, 62, 63, 64]. These findings are compatible with transneuronal degeneration and diaschisis after sensory deafferentation [65, 66], suggesting that reduced retinal input may contribute to visual cortical atrophy.

In parallel, functionally we observed a significant INT shortening within the DMN, a high‐order integrative system that typically shows longer intrinsic timescales than sensory and most association cortices [67, 68, 69]. In the context of reduced visual input, this shortening may suggest altered temporal organization in higher‐order cortical systems, although the biological basis of INT changes remains indirect. This interpretation is broadly consistent with prior resting‐state studies showing altered DMN–VN coupling and other DMN‐related functional changes across ophthalmic conditions, including glaucoma, RRD, and thyroid‐associated ophthalmopathy [70, 71, 72].

Along the macroscale hierarchy, RRD showed a downward shift of SCN Gradient 1 in the visual network and an upward shift in the limbic network. Given that Gradient 1 indexes the principal sensory‐to‐transmodal axis of cortical organization [27, 73], this bidirectional displacement may reflect altered macroscale cortical organization under visual deafferentation. Although gradient studies in ophthalmic disorders are limited, cross‐disorder work indicates that disruption of sensory inputs or transmodal hubs can systematically reshape this axis, often compressing sensory–transmodal differentiation [74, 75, 76]. Thus, the “visual‐downward and limbic‐upward” pattern in RRD may reflect system‐level alterations under visual deafferentation rather than an isolated visual‐cortical abnormality.

Importantly, these findings should be interpreted with caution. Similar cortical structural and functional alterations have also been reported in other visual deprivation‐related conditions, including diabetic retinopathy [77], age‐related macular degeneration [78], amblyopia [79], and cataract‐related visual impairment [80, 81]. Therefore, the abnormalities observed in the present study may not be entirely specific to RRD, but may partly reflect shared neurobiological consequences of reduced visual input [82]. At the same time, RRD is characterized by a relatively abrupt disruption of retinal input, which may provide a clinically informative model for studying cortical responses to sudden visual deafferentation. Nevertheless, because the present study was cross‐sectional and did not include disease‐control groups, we could not determine the extent to which these alterations were specifically related to RRD versus more general effects of visual deprivation.

Our neuroimaging–transcriptomic analysis suggested that the PLS2 component linked to SCN Gradient 1 was enriched for growth factor response, presynaptic/trans‐synaptic signaling, negative regulation of protein metabolism, and cholesterol biosynthesis. Growth factor–related programs are typically engaged after retinal injury and may reflect reactive cortical responses to abrupt deafferentation [83, 84]. Enrichment of synaptic signaling pathways is also consistent with prior evidence linking synaptic dysfunction to RRD and its neural sequelae [85, 86, 87, 88]. In addition, the involvement of protein metabolic regulation and cholesterol biosynthesis may point to metabolic processes relevant to synaptic integrity, given the established role of cholesterol in membrane organization, neurotransmitter release, and spine stability [89, 90], and is broadly consistent with reports linking cholesterol dysregulation to retinal degeneration and central neuroinflammation [91, 92]. However, because these associations were derived from population‐level reference atlases, they should be interpreted as indirect and exploratory rather than as direct evidence of specific molecular mechanisms in RRD.

Cell‐type decoding of the PLS2 component linked to SCN Gradient 1 suggested enrichment for excitatory neurons (Neuro‐Ex), inhibitory neurons (Neuro‐In), and microglia (Micro), providing population‐level cellular context for the observed transcriptomic associations. These signatures may be broadly consistent with synaptic, developmental, and immune‐related processes reported in prior literature [93, 94, 95]. In parallel, Gradient 1 abnormalities exhibited significant positive spatial correspondence with serotonergic (5‐HT1a, 5‐HT4, SERT), dopaminergic (D1, D2), VAChT, and MU receptor density maps, with minimal association to GABAa. Given that these neuromodulatory systems have been implicated in cortical excitation–inhibition balance, plasticity, and large‐scale dynamics [41, 96, 97], the combined cellular and neurochemical signatures may provide biologically informative context for SCN Gradient 1 abnormalities observed in RRD, although such atlas‐based associations remain indirect.

We evaluated five machine‐learning algorithms with SHAP interpretability using GMV and INT features. INT_LH_Default_Mode_Network showed the strongest discriminative value, with SVM achieving the best performance (AUC = 0.753), and SHAP consistently ranking this feature as the top contributor across models. These results suggest that INT, as a functional metric reflecting temporal features of neural activity [20, 21], may be more sensitive than GMV to RRD‐related changes in higher‐order systems such as the DMN. Prior work has also leveraged INT for accurate classification in adolescent major depressive disorder [98], supporting its feasibility as an auxiliary discriminative marker in RRD.

## 5 Limitations

Several limitations should be acknowledged. First, this single‐center cross‐sectional study with a modest sample size limits generalizability and precludes direct inference on longitudinal trajectories. In addition, because we did not include disease‐control groups with other forms of visual impairment, we were unable to distinguish RRD‐related effects from more general consequences of reduced visual input, nor could we establish the RRD‐specificity of the observed cortical alterations. These factors may also have contributed to the nonsignificant INT–GMV association. INT and SCN gradients should therefore be interpreted primarily as systems‐level imaging phenotypes, and their direct biological relevance to RRD pathology remains to be established. Similarly, the imaging‐transcriptomic and neurotransmitter mapping results should be regarded as spatial association findings that provide biologically informed context rather than confirmatory evidence of molecular or neurochemical mechanisms. Likewise, the machine‐learning results indicate discriminative utility but do not establish pathophysiological specificity or mechanism. Second, SCN gradients were derived from group‐level GMV covariance and may not translate to single‐subject features for supervised classification; future work should develop individualized SCN representations. Third, MR analyses relied on European‐ancestry GWAS and a relatively liberal instrument threshold (*p* < 5 × 10^−6^), which may introduce population mismatch and weak‐instrument bias despite sensitivity analyses, warranting validation in larger ancestry‐matched datasets. Fourth, transcriptomic mapping based on the AHBA is left‐hemisphere dominant; restricting analyses to the left hemisphere improves sampling consistency but may underestimate bilateral effects, and postmortem‐to‐in vivo correspondence remains an inherent approximation. Fifth, neurotransmitter receptor templates from healthy populations provide normative spatial references rather than participant‐specific neurochemistry, so findings should be interpreted as population‐level spatial correspondence. Sixth, although we applied repeated nested cross‐validation, pipeline‐based preprocessing, and permutation testing to reduce overfitting risk, the relatively modest sample size and absence of external validation may still limit the robustness and generalizability of the machine‐learning findings. Accordingly, the classification and SHAP‐based interpretability results should be considered exploratory rather than definitive. Finally, the absence of postoperative or longitudinal rescanning prevents direct testing of the “deafferentation–plasticity–recovery” trajectory, motivating prospective paired pre/post and longitudinal designs.

## 6 Conclusion

In summary, RRD is associated with coordinated structural and functional alterations across cortical hierarchies. This multiscale pattern is characterized by visual‐network atrophy, altered intrinsic neural timescales in higher‐order systems, and a redistribution of the principal SCN gradient under abrupt visual deafferentation. Exploratory imaging‐transcriptomic and neurotransmitter spatial association analyses may provide biological context for these abnormalities and inform future studies of prognosis and underlying mechanisms.

## Author Contributions

Y.J. drafted the initial manuscript and performed data visualization and statistical analyses; X.H. was responsible for data processing and quality control; Y.‐Y.W. contributed to data curation; L.Z. assisted with the literature search and review; Z.‐E.D. assisted with data interpretation; X.‐R.W. conceived and oversaw the study, provided methodological and clinical guidance, and critically revised the manuscript. All authors have read and approved the final version of the manuscript.

## Funding

We gratefully acknowledge the support of the National Natural Science Foundation of China (82160207), the Key Projects of Jiangxi Youth Science Fund (20202ACBL216008), the Science and Technology Plan of the Jiangxi Provincial Health and Health Commission (202130156), and the Graduate Student Research Project of Jiangxi Province (2025KXJYS601).

## Ethics Statement

This study was approved by the Ethics Committee of the First Affiliated Hospital of Nanchang University (Approval No. IIT [2024] Ethics No. 790). All procedures were conducted in accordance with the Declaration of Helsinki and relevant institutional guidelines and regulations. Written informed consent to participate was obtained from all participants.

## Conflicts of Interest

The authors declare no conflicts of interest.

## Supporting information


**Figure S1:** Performance of ML models using INT and GMV features to distinguish RRD patients from HCs, and corresponding SHAP‐based interpretations.
**Table S1:** Scanning parameters for BOLD sequences and structural T1‐weighted images.
**Table S2:** Information about the six donors in AHBA.
**Table S3:** Demographic and clinical features of RRD and HCs.
**Table S4:** Brain regions showing altered GMV and INT in patients with RRD compared with HCs.
**Table S5:** Mendelian randomization (MR) results. (A) Genetic instruments used in forward MR analyses (retinal detachment → GMV) after LD pruning and harmonization. (B) Genetic instruments used in reverse MR analyses (GMV → retinal detachment) after LD pruning and harmonization. (C) Causal effect estimates for forward MR analyses (retinal detachment → GMV) across MR methods. (D) Causal effect estimates for reverse MR analyses (GMV → retinal detachment) across MR methods. (E) Sensitivity analyses for forward MR (retinal detachment → GMV). (F) Sensitivity analyses for reverse MR (GMV → retinal detachment).
**Table S6:** PLS‐derived gene lists associated with SCN Gradient 1 abnormalities. (A) PLS2+ genes and weights (positively weighted genes; *Z* > 0). (B) PLS2− genes and weights (negatively weighted genes; *Z* < 0).
**Table S7:** Classification performance of models using GMV and INT feature sets to distinguish RRD from HCs.
**Appendix S1:** Supplementary methods.

## Data Availability

The raw data supporting the conclusions of this article are available from the corresponding author upon reasonable request. Requests to access the datasets should be directed to Xiao‐Rong Wu, ndyfy03457@ncu.edu.cn. The acquisition of MRI data for this study was undertaken at the Jiangxi Provincial Medical Imaging Clinical Research Center/Clinical Research Center for Medical Imaging in Jiangxi Province (Registration No. 20223BCG74001).
