# Supplementary material for: Multiscale Cortical Remodeling Following Abrupt Visual Deafferentation in Rhegmatogenous Retinal Detachment: Imaging Transcriptomics and Neurotransmitter Mapping
Source: CNS Neurosci Ther. 2026 May 13;32(5):e70925. doi: 10.1002/cns.70925 (PMC13170476; doi:10.1002/cns.70925)
Supplement: Supplementary file 1 — Figure S1: Performance of ML models using INT and GMV features to distinguish RRD patients from HCs, and corresponding SHAP‐based interpretations. Table S1: Scanning parameters for BOLD sequences and structural T1‐weighted images. Table S2: Information about the six donors in AHBA. Table S3: Demographic and clinical features of RRD and HCs. Table S4: Brain regions showing altered GMV and INT in patients with RRD compared with HCs. Table S5: Mendelian randomization (MR) results. (A) Genetic instruments used in forward MR analyses (retinal detachment → GMV) after LD pruning and harmonization. (B) Genetic instruments used in reverse MR analyses (GMV → retinal detachment) after LD pruning and harmonization. (C) Causal effect estimates for forward MR analyses (retinal detachment → GMV) across MR methods. (D) Causal effect estimates for reverse MR analyses (GMV → retinal detachment) across MR methods. (E) Sensitivity analyses for forward MR (retinal detachment → GMV). (F) Sensitivity analyses for reverse MR (GMV → retinal detachment). Table S6: PLS‐derived gene lists associated with SCN Gradient 1 abnormalities. (A) PLS2+ genes and weights (positively weighted genes; Z > 0). (B) PLS2− genes and weights (negatively weighted genes; Z < 0). Table S7: Classification performance of models using GMV and INT feature sets to distinguish RRD from HCs. Appendix S1: Supplementary methods. [file CNS-32-e70925-s001.zip › Supinfo.docx]

**Supporting Information：**

**Multiscale Cortical Remodeling Following Abrupt Visual Deafferentation in Rhegmatogenous Retinal Detachment: Imaging Transcriptomics and Neurotransmitter Mapping**

# **Yu Ji^1*^, Xin Huang^234*^, Yuan-Yuan Wang^5*^, Lin Zhou^1^, Zhuo-Er Dong^1^, Xiao-Rong Wu^1#^**

1.Department of Ophthalmology, The First Affiliated Hospital, Jiangxi Medical College, Nanchang University, Nanchang 330006 Jiangxi Province, China

2.The Affiliated Eye Hospital, Jiangxi Medical College, Nanchang University, Nanchang 330006, Jiangxi, China.

3.Jiangxi Province Key Laboratory of Ophthalmology and Vision Sciences, Nanchang 330006,Jiangxi, China.

4.Jiangxi Clinical Research Center for Ophthalmic Disease, Nanchang 330006, Jiangxi, China.

5.Department of Radiology, The First Affiliated Hospital, Jiangxi Medical College, Nanchang University, Nanchang 330006 Jiangxi Province, China

#Corresponding author: Xiao-Rong Wu, MD, PHD. Department of Ophthalmology, The First Affiliated Hospital, Jiangxi Medical College, Nanchang University, Nanchang 330006 Jiangxi Province, China; Tel: +86 13617093259, E-mail address: ndyfy03457@ncu.edu.cn.

*These authors contributed equally to this work

**Supplementary Methods.**

**Participants**

The study was approved by the Ethics Committee of the First Affiliated Hospital of Nanchang University (Approval No. IIT [2024] Ethics No. 790), and written informed consent was obtained.

RRD diagnosis was independently confirmed by two experienced retinal specialists using fundus examination, OCT, and B-scan ultrasonography, in accordance with established clinical guidelines. Inclusion criteria were: (1) spontaneous retinal detachment with at least one identifiable retinal break; (2) involvement of one or more retinal quadrants; and (3) absence of other ocular abnormalities in either eye. Exclusion criteria included RRD secondary to high myopia or trauma, diabetic retinopathy, a history of vitreoretinal surgery, or systemic diseases such as cardiovascular, neuropsychiatric, or cerebrovascular disorders.

HCs underwent comprehensive ophthalmologic and neurological screening and were required to meet the following criteria: (1) no history of ocular or systemic disease; (2) BCVA ≥ 1.0; and (3) normal findings on MRI, OCT, and B-scan ultrasonography.

**MRI Data Acquisition**

MRI scans were performed on a 3.0 T Siemens Trio Tim scanner (Siemens Healthcare, Erlangen, Germany) using an eight-channel phased-array head coil at the First Affiliated Hospital of Nanchang University. Each participant was positioned comfortably in the scanner in a supine posture with eyes gently closed. They were asked to remain motionless, relaxed, and awake throughout the acquisition, avoiding intentional cognitive engagement. Wakefulness was continuously monitored by a real-time sleep surveillance system. To minimize head motion and acoustic noise, foam padding was used to secure the head and earplugs were provided for noise attenuation. The detailed imaging parameters for the resting-state fMRI sequence are summarized in Table S1.

**VBM Analysis**

VBM analysis was performed using the Computational Anatomy Toolbox (CAT12; https://neuro-jena.github.io/cat/) implemented in Statistical Parametric Mapping software (SPM12; https://www.fil.ion.ucl.ac.uk/spm/) running on the MATLAB R2022b platform (MathWorks, Natick, MA, USA).

First, all T1-weighted structural images were converted from DICOM to NIFTI format to ensure compatibility with subsequent preprocessing steps. Each image was then visually inspected and subjected to automated quality control to exclude scans affected by motion artifacts or poor image quality. Next, image registration and tissue segmentation were performed using the New Segment and DARTEL algorithms to generate gray matter, white matter, and cerebrospinal fluid (CSF) maps. The segmented images were subsequently normalized to the Montreal Neurological Institute (MNI) space and resampled to a voxel resolution of 1.5 × 1.5 × 1.5 mm³. Finally, the normalized GM images were smoothed with an 8 mm full-width at half-maximum (FWHM) Gaussian kernel to enhance the signal-to-noise ratio and satisfy the assumptions of Gaussian random field theory.

For group-level statistical comparisons, independent two-sample t-tests were conducted to examine differences in GMV between RRD patients and HCs, with age, sex, and total intracranial volume (TIV) included as covariates. Statistical significance was determined using Gaussian random field (GRF) correction, with thresholds set at a voxel-wise *p* < 0.01 and cluster-level *p* < 0.01.

**MR Analysis**

Previous studies have suggested that structural damage to the retina may be closely associated with morphological alterations in the brain^1^. To further minimize the influence of potential confounders such as environmental factors, lifestyle, and genetic background, and to validate the neuroimaging findings, a two-sample MR approach was employed to investigate the causal relationship between retinal detachment–related phenotypes (exposures) and GMV (outcome).

Exposure data were obtained from the FinnGen consortium (R9 release, European ancestry) and included four phenotypes: retinal breaks without detachment, retinal detachment with breaks, retinal detachment and breaks, and unspecified or serous retinal detachment. Outcome data were derived from the UK Biobank (UKB) neuroimaging dataset described, which comprised 33,224 participants of European descent^2^.

Single nucleotide polymorphisms (SNPs) significantly associated with the exposure phenotypes at the genome-wide level (*p* < 5 × 10⁻⁶) were selected as candidate instrumental variables (IVs). The strength of each IV was evaluated using the F-statistic (F > 10). Five complementary MR methods were applied to estimate the causal effects: inverse variance weighting method under a multiplicative random-effects model (IVW-MRE), MR-Egger regression, weighted median, simple mode, and weighted mode.

In sensitivity analyses (significance threshold set at 0.1), Cochran’s Q test was used to assess heterogeneity among IVs, and the MR-Egger intercept test was employed to detect potential horizontal pleiotropy. In addition, the MR-PRESSO method was used to identify and correct outlier SNPs.

All MR analyses were performed in R software (version 4.2.1) using the “TwoSampleMR” (version 0.5.6) and “MRPRESSO” packages. A two-sided *p* < 0.05 was considered statistically significant.

**INT Analysis**

Resting-state fMRI data were preprocessed using Statistical Parametric Mapping software (SPM12; https://www.fil.ion.ucl.ac.uk/spm/) and the RESTplus_v1.28 toolbox^3^(http://www.restfmri.net/forum/RESTplus) implemented on the MATLAB R2022b platform (MathWorks, Natick, MA, USA). The estimation of INT followed previously established methods that quantify the temporal autocorrelation properties of the blood-oxygen-level-dependent (BOLD) signal^4,5^, in which the temporal decay characteristics of local BOLD autocorrelation are converted into timescale indices. Preprocessing steps included: (1) converting functional images from DICOM to NIFTI format; (2) discarding the initial time points; (3) slice-timing correction; (4) head motion correction; (5) spatial normalization; and (6) spatial smoothing with a 6 × 6 × 6 mm³ FWHM Gaussian kernel.

Subsequently, within the Schaefer 400-parcel cortical mask (7-network parcellation), the BOLD time series of each voxel was extracted and mean-centered. The temporal autocorrelation structure of each voxel’s time series was then computed. Specifically, the normalized autocorrelation function (ACF) was calculated across time lags ranging from 0 to 6 repetition times (TRs; TR = 2 s). The ACF reflects the similarity between a signal and its time-shifted version at different lags, thereby describing how long local BOLD activity remains temporally dependent on itself.

To obtain the INT value for each voxel, we quantified the area under the initial positive segment of its ACF. Specifically, starting from lag 0, the positive ACF values were cumulatively summed along the time-lag axis until the first nonpositive value (ACF ≤ 0) was reached. The sum of these positive ACF values was then multiplied by the TR (2 s) to yield the INT for that voxel, expressed in seconds. Formally, for voxel *v*, the INT is defined as follows:

$$\text{INT}(v)=\text{TR}\times\sum_{k=0}^{k^{*}} \text{ACF}_{v}(k),$$

where $\text{ACF}_{v}(k)$ denotes the normalized autocorrelation value of voxel *v* at lag *k*, and $k^{*}$ represents the first lag at which ACF ≤ 0. This index quantifies the temporal persistence of local BOLD signal autocorrelation, reflecting the intrinsic integration window of neural activity. Larger INT values indicate longer temporal persistence of regional activity, whereas smaller values represent faster fluctuations.

Based on the above procedure, whole-brain INT maps were generated for each participant. Group-level comparisons between RRD patients and HCs were conducted using two-sample t-tests, with multiple comparison correction performed using GRF correction (voxel-level *p* < 0.01, cluster-level *p* < 0.01, two-tailed).

**SCN Gradient Calculation**

The SCN gradient was computed following established analytical frameworks from previous studies^6^. To characterize large-scale patterns of structural co-organization, group-level structural covariance matrices were constructed separately for the RRD and HCs. Specifically, sex, age, and mean GMV were included as covariates and regressed out from each brain region’s GMV values. Partial Pearson correlation coefficients between every pair of brain regions were then calculated using the adjusted GMV data to generate the group-level structural covariance matrix. The resulting correlation matrices were Fisher z-transformed and subsequently entered into gradient decomposition analysis using the BrainSpace toolbox (https://github.com/MICA-MNI/BrainSpace)^7^.

The gradient decomposition procedure comprised the following steps: (1) applying a 90% sparsity threshold to each column of the z-transformed covariance matrix; (2) converting the thresholded matrix into an affinity matrix using a normalized angle similarity kernel to quantify the similarity of covariance profiles between regions; and

(3) performing nonlinear dimensionality reduction via diffusion embedding with a density normalization parameter of α = 0.5 to control for potential biases arising from variations in sampling density^8,9^.

To ensure cross-group comparability, the gradient spaces were aligned using Procrustes rotation. Specifically, a unified gradient template was generated based on the HC group and subsequently used to constrain gradient orientation in both RRD and HC groups, thereby minimizing disease-related bias in gradient alignment. Furthermore, the group-level gradient derived from the HCs in this study was compared with that obtained from healthy adults in the Human Connectome Project (HCP) to verify the reproducibility and biological validity of the gradient patterns.

**Gene Expression Data Preprocessing**

The gene expression data used in this study were obtained from the AHBA (http://human.brain-map.org), which contains postmortem brain samples from six adult donors, comprising a total of 3,702 sampling sites^10^. To ensure spatial correspondence with our neuroimaging findings, transcriptomic data were processed using the abagen toolbox (https://www.github.com/netneurolab/abagen)^11^, and the resulting gene expression profiles were mapped onto the Schaefer 400 cortical parcellation template.

Following the standardized pipeline established in previous studies^12^, preprocessing of the gene expression data included the following steps: (1) updating probe-to-gene annotations; (2) filtering probes based on signal intensity to remove those with low expression or low reliability; (3) selecting a representative probe for each gene when multiple probes were available; (4) assigning transcriptomic samples to their corresponding cortical parcels based on spatial coordinates; (5) addressing missing data to mitigate sampling bias; (6) normalizing expression values across samples; (7) normalizing across genes to improve inter-donor comparability; (8) computing weighted averages between samples and regions to obtain stable regional expression estimates; and (9) retaining only genes with consistent expression profiles across donors to ensure robustness and reproducibility.

After preprocessing, 15,633 genes were retained for further analyses. It is important to note that only two of the six AHBA donors included right-hemisphere sampling, while the remaining four provided left-hemisphere data only. To ensure spatial consistency and statistical reliability, the present analyses were therefore restricted to the left hemisphere. Detailed demographic and sampling information for the six donors is provided in Supplementary Table S2.

Based on this procedure, we obtained a region-by-gene expression matrix consisting of 200 left-hemisphere cortical parcels (from the Schaefer 400 atlas) and 15,633 genes, which served as the input for subsequent neuroimaging–transcriptomic integration analyses.

**Transcription–Neuroimaging Association Analysis**

Partial least squares (PLS) regression^13^ was employed to examine the spatial correspondence between gene expression patterns and abnormalities in the SCN gradient. Specifically, the z-score–standardized gene expression matrix from 200 left-hemisphere cortical parcels (200 × 15,633 genes) was used as the predictor variable, while the z-score–standardized group difference map of SCN gradient 1 (RRD versus HCs; 200 × 1 t-values) served as the response variable. PLS projects high-dimensional transcriptomic data onto a set of orthogonal latent components that maximize the covariance between gene expression and neuroimaging features, with components ranked according to the proportion of explained covariance^14^. In the present analysis, the second partial least squares component (PLS2)—rather than the first partial least squares component (PLS1)—accounted for the largest proportion of the shared variance; therefore, PLS2 was considered the principal component and used for subsequent biological interpretation. To assess the statistical significance of this component, a spin permutation test preserving spatial autocorrelation was performed with 10,000 permutations, evaluating whether the observed correspondence exceeded that expected by chance. Additionally, gene weights associated with the main component were subjected to the same permutation-based significance testing, and genes surviving false discovery rate (FDR) correction (*p* < 0.05) were retained for subsequent functional enrichment analyses.

**Gene Enrichment Analysis**

Functional enrichment analysis of significant genes was performed using Metascape (https://metascape.org/gp/index.html#/main/step1)^15^. Specifically, Gene Ontology (GO) annotations were applied across three major domains—biological process (BP), molecular function (MF), and cellular component (CC)—to elucidate the potential biological mechanisms associated with the principal PLS component. Enrichment significance was determined at *p* < 0.05, with multiple comparison correction using the FDR method. To further distinguish the functional relevance of transcriptional patterns in opposite directions, enrichment analyses were independently conducted for the positively weighted gene set (PLS2⁺) and the negatively weighted gene set (PLS2⁻).

**Cell Type Analysis**

Following established strategies for cell-type analysis^16^, we integrated results from five large-scale single-cell transcriptomic studies of the human cerebral cortex to obtain comprehensive lists of cell type–specific gene sets, representing the molecular profiles of major cortical cell lineages^17^. Based on this integrated reference, seven core brain cell types were extracted: astrocytes (Astro), endothelial cells (Endo), microglia (Micro), excitatory neurons (Neuro-Ex), inhibitory neurons (Neuro-In), oligodendrocytes (Oligo), and oligodendrocyte precursor cells (OPCs). The key gene sets identified from the PLS analysis that were associated with RRD were then compared with each cell type–specific gene set to infer the cellular specificity of these genes. To determine whether the observed overlaps exceeded those expected by chance, permutation testing was performed to generate a null distribution, and a p-value was computed for each cell type. The resulting *p*-values were corrected for multiple comparisons using the FDR method, with a significance threshold set at *p* < 0.001.

**Spatial Correlation with Neurotransmitter Density Maps**

To evaluate whether alterations in SCN gradient 1 observed in RRD patients were associated with specific neurotransmitter systems, spatial correlation analyses were performed between neuroimaging-derived difference maps and molecular imaging atlases using the JuSpace toolbox (https://github.com/juryxy/JuSpace)^18^. A total of 44 PET/SPECT–based molecular imaging maps were included, encompassing a wide range of classical neurotransmitter receptors, transporters, and related molecular targets.

Specifically, the group difference map of SCN gradient 1 (expressed as t-values for RRD relative to HCs) was used as the neuroimaging phenotype. This map was then spatially correlated with each PET/SPECT-derived molecular map to assess whether the spatial distribution of SCN gradient 1 abnormalities corresponded to that of particular neurotransmitter systems, thereby revealing their potential neurochemical substrates.

To determine the statistical significance and robustness of these spatial correlations, 1,000 permutation tests were performed for each “neuroimaging–molecular map” pair, generating null distributions through spatial randomization to evaluate whether the observed correlations exceeded those expected by chance. Finally, *p*-values from all 44 molecular maps were corrected for multiple comparisons using the FDR method, and only neurotransmitter systems that survived FDR correction were considered to be significantly associated with SCN gradient 1 abnormalities.

**Machine Learning Analysis**

To evaluate the discriminative ability of GMV and INT features in distinguishing RRD patients from HCs, we extracted all GMV and INT measures that showed significant group differences as input variables for model construction. The SCN gradient was not included because it is derived from a dimensionality reduction of the group-level structural covariance network, representing a shared macroscale topological organization across participants—i.e., a population-level low-dimensional representation rather than an individual-level feature vector. Consequently, SCN gradients are not directly assignable to individual subjects and thus unsuitable for supervised classification modeling.

Five supervised classifiers were implemented to construct binary classification models: SVM, RF, LR, XGBoost, and LightGBM. All models were implemented and trained in the Python environment using a unified analytical pipeline. Specifically, z-score normalization was applied to the training set, and the same transformation was subsequently applied to the corresponding test set to avoid data leakage. Hyperparameter tuning for each model was performed using grid search optimization.

Model performance was assessed using repeated nested cross-validation. The outer loop employed 10-fold stratified cross-validation, repeated 10 times to preserve class balance and improve robustness, while the inner loop used 5-fold grid search to identify optimal hyperparameters based on the area under the receiver operating characteristic curve (AUC). The optimized model was then applied to the outer test folds to generate class probability estimates independent of the training process. From these outer test results, we calculated accuracy, AUC, sensitivity, specificity, precision, and F1 score, and reported their mean values across all folds and repetitions as the overall classification performance. Receiver operating characteristic (ROC) curves were also generated based on the aggregated predictions to visually depict classification ability.

To further determine whether the model’s discriminative performance exceeded chance levels, a nonparametric permutation test was conducted. Specifically, class labels were randomly shuffled while keeping the feature matrix unchanged, and the entire training–validation process was repeated 5,000 times to construct an empirical null distribution of AUC values. The observed mean AUC from the true labels was then compared with this null distribution to compute the permutation *p*-value, thereby assessing the statistical significance of the model’s discriminative performance.

**SHAP Analysis**

To enhance the interpretability of the classification models and identify the most discriminative neuroimaging features, SHAP values were computed for each optimized classifier within the Python environment. SHAP is grounded in cooperative game theory and quantifies the marginal contribution of each feature to the model’s output by averaging its effect across all possible feature combinations. This approach allows the estimation of both the magnitude and direction of each feature’s influence on the predicted probability of being classified as an RRD patient. In practical implementation, TreeExplainer was applied to tree-based models (RF, XGBoost, and LightGBM), whereas KernelExplainer was used for non-tree models (SVM and LR), ensuring methodological consistency and stable estimation of feature contributions across model architectures. This framework enables model outputs to be decomposed into specific GMV and INT features, thereby delineating—at both the individual and group levels—which structural and functional attributes predominantly drive the classification of RRD.

**Statistical Analysis**

The normality of demographic and clinical variables was first assessed using the Shapiro–Wilk test. For variables following a normal distribution, independent-sample t-tests were used for group comparisons; for non-normally distributed variables, the Mann–Whitney U test was applied. Chi-square tests were used to compare categorical variables. Group-level comparisons of demographic and clinical characteristics were performed using SPSS version 27 (SPSS Inc., Chicago, IL, USA).

Group differences in INT and GMV were evaluated using voxel-wise two-sample t-tests, with multiple comparison correction performed using the GRF method (voxel-level *p* < 0.01, cluster-level *p* < 0.01, two-tailed). For SCN gradient comparisons, t-tests were conducted at the level of the Yeo 7-network parcellation, and multiple comparisons were controlled using the Benjamini–Hochberg FDR correction (*p* < 0.05, two-tailed).

To account for potential confounding effects, age, sex, and years of education were included as covariates in all neuroimaging analyses. Covariate adjustment was performed to enhance the validity and robustness of the statistical findings.

**Figures**


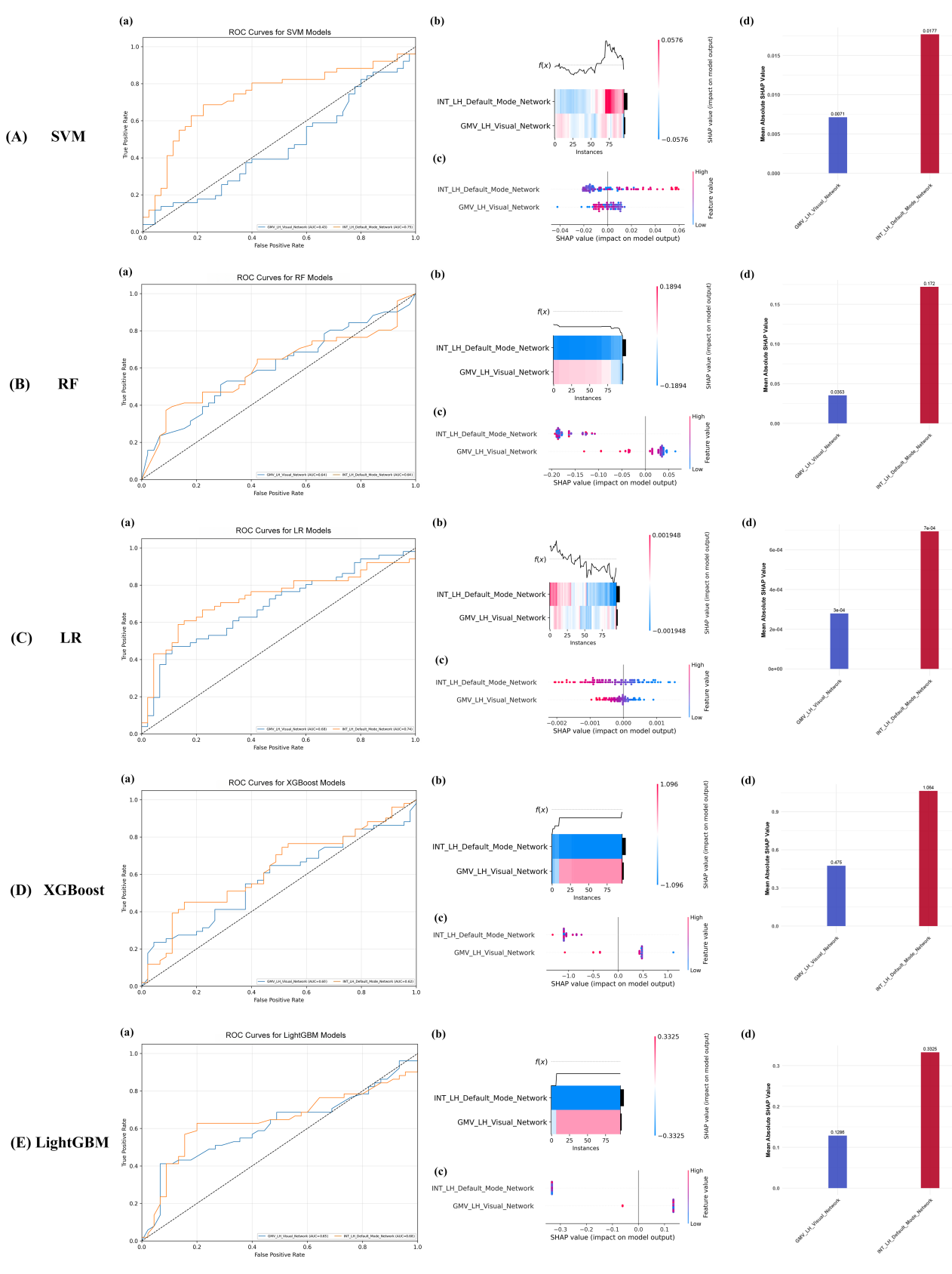


**Supplementary Figure S1**. Performance of ML models using INT and GMV features to distinguish RRD patients from HCs, and corresponding SHAP-based interpretations.

(A–E) Results of the five ML classifiers—SVM, RF, LR, XGBoost, and LightGBM—and their respective SHAP interpretations:

(a) ROC curves of each ML model; (b) SHAP decision/contribution trajectories; (c) SHAP summary (beeswarm) plots; (d) Bar plots showing the mean absolute SHAP values of all features.

Abbreviations: INT, intrinsic neural timescale; GMV, gray matter volume; RRD, rhegmatogenous retinal detachment; HCs, healthy controls; SVM, support vector machine; RF, random forest; LR, logistic regression; XGBoost, eXtreme gradient boosting; LightGBM, light gradient boosting machine; SHAP, SHapley Additive exPlanations; AUC, area under the ROC curve; ROC, receiver operating characteristic.

**Tables**

| **Table S1**. Scanning parameters for BOLD sequences and structural T1-weighted images   \| scanning parameters \| 3D-T1 \| EPI \| \| --- \| --- \| --- \| \| TR (ms) \| 1900 \| 2000 \| \| TE (ms) \| 2.26 \| 30 \| \| FOV (mm^2^) \| 256 x 256 \| 200 x 200 \| \| matrix \| 256 x 256 \| 64 x 64 \| \| Slice thickness (mm) \| 1 \| 4 \| \| Interslice gap (mm) \| 0.5 \| 1.2 \|   Abbreviations: TR, repetition time; TE, echo time; FOV, field of view; BOLD, blood-oxygen-level-dependent. |
| --- | --- | --- | --- | --- | --- | --- | --- | --- | --- | --- | --- | --- | --- | --- | --- | --- | --- | --- | --- | --- | --- |

| **Table S2**. Information about the six donors in AHBA. | | | | | |
| --- | --- | --- | --- | --- | --- |
| **Donor** | **Age** | **Sex** | **Ethnicity** | **Hemisphere** | **Post-mortem interval ^a^** |
| H0351 1009 | 57 | Male | Caucasian | L | 25.5 h |
| H0351 1012 | 31 | Male | Caucasian | L | 17.5 h |
| H0351 1015 | 49 | Female | Hispanic | L | 30 h |
| H0351 1016 | 55 | Male | Caucasian | L | 18 h |
| H0351 2001 | 24 | Male | African American | L + R | 23 h |
| H0351 2002 | 39 | Male | African American | L + R | 10 h |
| ^a^ Post-mortem interval is defined as the time period from the time of death to the time the tissue is frozen.  Abbreviations: AHBA, Allen Human Brain Atlas; L, left; R, Right. | | | | | |

**Table S3**. Demographic and Clinical Features of RRD and HCs

|  | **RRD (n=51)** | **HCs (n=45)** | **Statistic** | ***p* value** |
| --- | --- | --- | --- | --- |
| Men/women | 23/28 | 18/27 | χ² = 0.254 | 0.614^a^ |
| Age (years)  median (IQR) | 53 (42, 64) | 54 (45.5, 60.5) | U = 1092.500 | 0.686^b^ |
| Duration of detachment (days)  median (IQR) | 15 (7, 30) | N/A | N/A | N/A |
| IOP (mmHg)  median (IQR) | 15 (12, 16.75) | N/A | N/A | N/A |
| Vision  median (IQR) | 0.040 (0.010, 0.143) | N/A | N/A | N/A |
| Axial length of eye (mm)  (mean ± SD) | 24.700 ± 2.058 | N/A | N/A | N/A |
| HAMA score  median (IQR) | 4 (2, 6) | N/A | N/A | N/A |

Abbreviations: RRD, Rhegmatogenous Retinal Detachment; HCs, Healthy Controls; HAMA, Hamilton Anxiety Scale; IOP, Intraocular Pressure; IQR, interquartile range; SD, standard deviation; N/A, not applicable; ^a^, Chi-square test; ^b^, Mann–Whitney U test.

**Table S4**. Brain regions showing altered GMV and INT in patients with RRD compared with HCs.

|  | **Brain network** | **Peak t value** | **MNI coordinates**  **(x, y, z)** | **Cluster size**  **(voxels)** |
| --- | --- | --- | --- | --- |
| GMV | LH_Visual_Network | -3.766 | -4.5 -94.5 0 | 7294 |
| INT | LH_Default_Mode_Network | -4.302 | -57 0 -9 | 1141 |

Abbreviations:RRD, rhegmatogenous retinal detachment; HCs, healthy controls; GMV, gray matter volume; INT, intrinsic neural timescale; VN, visual network; DMN, default mode network; LH, left hemisphere; MNI, Montreal Neurological Institute; GRF, Gaussian random field. Statistical threshold: voxel-level *p* < 0.01 and cluster-level *p* < 0.01 (two-tailed; GRF corrected).

**Table S5**. Mendelian randomization (MR) results (Excel file).

This Excel file contains (i) genetic instruments after LD pruning and harmonization for forward and reverse MR analyses, (ii) full causal effect estimates across MR methods, and (iii) sensitivity analyses.

**Table S5A**. Genetic instruments used in forward MR analyses (retinal detachment → GMV) after LD pruning and harmonization.

**Table S5B**. Genetic instruments used in reverse MR analyses (GMV → retinal detachment) after LD pruning and harmonization.

**Table S5C**. Causal effect estimates for forward MR analyses (retinal detachment → GMV) across MR methods.

**Table S5D**. Causal effect estimates for reverse MR analyses (GMV → retinal detachment) across MR methods.

**Table S5E**. Sensitivity analyses for forward MR (retinal detachment → GMV).

**Table S5F**. Sensitivity analyses for reverse MR (GMV → retinal detachment).

**Table S6**. PLS-derived gene lists associated with SCN Gradient 1 abnormalities (Excel file).

This Excel file provides the genes significantly associated with SCN Gradient 1 alterations based on the PLS regression linking the Gradient 1 case–control t-map to AHBA gene expression. For each gene, the file reports the gene name, gene ID, and standardized PLS weight (Z-score). The Excel file contains two sheets:

**Table S6A**. PLS2+ genes and weights (positively weighted genes; Z > 0).

**Table S6B**. PLS2− genes and weights (negatively weighted genes; Z < 0).

**Table S7**. Classification performance of models using GMV and INT feature sets to distinguish RRD from HCs

| Model | Feature set | AUC | Accuracy | Sensitivity | Specificity | Precision | F1 |
| --- | --- | --- | --- | --- | --- | --- | --- |
| SVM | GMV_LH_Visual_Network | 0.431 | 0.559 | 0.660 | 0.455 | 0.573 | 0.597 |
|  | INT_LH_Default_Mode_Network | 0.753 | 0.688 | 0.670 | 0.700 | 0.766 | 0.691 |
| RF | GMV_LH_Visual_Network | 0.643 | 0.603 | 0.630 | 0.575 | 0.643 | 0.627 |
|  | INT_LH_Default_Mode_Network | 0.635 | 0.633 | 0.607 | 0.655 | 0.710 | 0.636 |
| LR | GMV_LH_Visual_Network | 0.680 | 0.621 | 0.700 | 0.535 | 0.603 | 0.636 |
|  | INT_LH_Default_Mode_Network | 0.739 | 0.674 | 0.723 | 0.615 | 0.718 | 0.698 |
| XGBoost | GMV_LH_Visual_Network | 0.601 | 0.540 | 0.590 | 0.480 | 0.575 | 0.576 |
|  | INT_LH_Default_Mode_Network | 0.623 | 0.656 | 0.610 | 0.695 | 0.746 | 0.639 |
| LightGBM | GMV_LH_Visual_Network | 0.653 | 0.622 | 0.630 | 0.620 | 0.670 | 0.637 |
|  | INT_LH_Default_Mode_Network | 0.684 | 0.687 | 0.630 | 0.750 | 0.792 | 0.678 |

Abbreviations: RRD, Rhegmatogenous Retinal Detachment; HCs, Healthy Controls; GMV, gray-matter volume; INT, intrinsic neural timescale; LH, left hemisphere; DMN, Default Mode Network; VN, Visual Network; SVM, Support Vector Machine; RF, Random Forest; LR, Logistic Regression; XGBoost, eXtreme Gradient Boosting; LightGBM, Light Gradient Boosting Machine; AUC, area under the ROC curve; F1, F1-score.

**REFERENCES**

1. Li B, Liu Y, Li H, et al. Reduced gray matter volume in patients with retinal detachment: evidence from a voxel-based morphometry study. *Acta Radiol*. 2020;61(3):395-403.

2. Smith SM, Douaud G, Chen W, et al. An expanded set of genome-wide association studies of brain imaging phenotypes in UK Biobank. *Nat Neurosci*. 2021;24(5):737-745.

3. Jia X, Wang J, Sun H, et al. RESTplus: an improved toolkit for resting-state functional magnetic resonance imaging data processing. *Sci Bull (Beijing)*. 2019;64(14):953-954.

4. Wolff A, Berberian N, Golesorkhi M, Gomez-Pilar J, Zilio F, Northoff G. Intrinsic neural timescales: temporal integration and segregation. *Trends Cogn Sci*. 2022;26(2):159-173.

5. Wu K, Gollo LL. Mapping and modeling age-related changes in intrinsic neural timescales. *Commun Biol*. 2025;8(1):167.

6. Fan Y, Xu Y, Wan B, et al. Anterior-posterior systematic deficits of cortical thickness in early-onset schizophrenia. *Commun Biol*. 2025;8(1):778.

7. de Wael RV, Benkarim O, Paquola C, et al. BrainSpace: a toolbox for the analysis of macroscale gradients in neuroimaging and connectomics datasets. *Commun Biol*. 2020;3(1):103.

8. Margulies DS, Ghosh SS, Goulas A, et al. Situating the default-mode network along a principal gradient of macroscale cortical organization. *Proc Natl Acad Sci U S A*. 2016;113(44):12574-12579.

9. Paquola C, De Wael RV, Wagstyl K, et al. Microstructural and functional gradients are increasingly dissociated in transmodal cortices. *PLoS Biol*. 2019;17(5):e3000284.

10. Hawrylycz MJ, Lein ES, Guillozet-Bongaarts AL, et al. An anatomically comprehensive atlas of the adult human brain transcriptome. *Nature*. 2012;489(7416):391-399.

11. Markello RD, Arnatkeviciute A, Poline J, Fulcher BD, Fornito A, Misic B. Standardizing workflows in imaging transcriptomics with the abagen toolbox. *Elife*. 2021;10.

12. Arnatkeviciute A, Fulcher BD, Fornito A. A practical guide to linking brain-wide gene expression and neuroimaging data. *Neuroimage*. 2019;189:353-367.

13. Abdi H, Williams LJ. Partial least squares methods: partial least squares correlation and partial least square regression. *Methods Mol Biol*. 2013;930:549-579.

14. Krishnan A, Williams LJ, McIntosh AR, Abdi H. Partial Least Squares (PLS) methods for neuroimaging: a tutorial and review. *Neuroimage*. 2011;56(2):455-475.

15. Zhou Y, Zhou B, Pache L, et al. Metascape provides a biologist-oriented resource for the analysis of systems-level datasets. *Nat Commun*. 2019;10(1):1523.

16. Li J, Seidlitz J, Suckling J, et al. Cortical structural differences in major depressive disorder correlate with cell type-specific transcriptional signatures. *Nat Commun*. 2021;12(1):1647.

17. Seidlitz J, Nadig A, Liu S, et al. Transcriptomic and cellular decoding of regional brain vulnerability to neurogenetic disorders. *Nat Commun*. 2020;11(1):3358.

18. Dukart J, Holiga S, Rullmann M, et al. JuSpace: A tool for spatial correlation analyses of magnetic resonance imaging data with nuclear imaging derived neurotransmitter maps. *Hum Brain Mapp*. 2021;42(3):555-566.
